# Systemic Therapy for Early-Stage Breast Cancer: What the Plastic Surgeon Should Know

**Published:** 2017-02-21

**Authors:** Chad M. Teven, Daniel B. Schmid, Mark Sisco, James Ward, Michael A. Howard

**Affiliations:** ^a^Section of Plastic and Reconstructive Surgery, The University of Chicago Medicine, Chicago, Ill; ^b^Summit Medical Group, Millburn, NJ; ^c^Divisions of Plastic and Reconstructive Surgery; ^d^Hematology/Oncology, NorthShore University Health System, Evanston, Ill

**Keywords:** breast reconstruction, chemotherapy, early-stage breast cancer, hormonal therapy, targeted therapy

## Abstract

**Objective:** We review the types, indications, and common regimens of systemic forms of therapy offered in early-stage breast cancer. We further detail the mechanism of action, approved uses, major toxicities, and relevance to breast reconstruction of specific agents. **Methods:** A review of the literature on PubMed and Cochrane databases was undertaken to define the indications and common regimens of systemic therapy in early-stage breast cancer. In addition, literature describing relevant information regarding specific systemic agents was reviewed. **Results:** The main objectives of systemic therapy, when provided in the perioperative setting, are to reduce the risk for future recurrence and prolong overall survival. Systemic forms of therapy consist of chemotherapy, hormonal therapy, and targeted therapy and are increasingly being offered to women with early-stage breast cancer. Similarly, as more women are diagnosed with disease that is amenable to surgical extirpation, rates of breast reconstruction are on the rise. Many agents have effects that may impact patient safety with respect to breast reconstruction. **Conclusions:** Increasingly, women with breast cancer receive 1 or more forms of systemic therapy during the course of their treatment. It is therefore of significant importance that plastic surgeons have a clear understanding of the issues surrounding the use of systemic agents.

The American Cancer Society reported that in 2015, approximately 231,840 cases of invasive breast cancer and 50,041 cases of ductal carcinoma in situ were diagnosed in the United States.^1^ Breast cancer treatment can include surgery, radiotherapy, systemic modalities, or a combination thereof. In determining the optimal approach for the type, timing, and sequence of treatment, several variables are considered, including disease stage, genetic predisposition, functional status, medical history, social situation, and patient preference. In addition, a variety of prognostic and predictive disease markers that determine the clinicopathologic and molecular subtype of the disease are considered.^2^ For early-stage breast cancer (ie, stages IA, IB, IIA, IIB, and IIIA), in which disease is confined to the breast with or without spread to the locoregional lymph nodes, standard treatment includes surgical extirpation, and, in select patients, radiotherapy (Table 1). In addition, it is increasingly common for oncologists to offer systemic therapy to patients with early-stage breast cancer.

Systemic forms of therapy include chemotherapy, hormonal therapy, and targeted therapy. The decision to use systemic therapy depends on several factors, including menopause status, tumor size and behavior, and the presence and activity of specific receptors. While surgery and radiation therapy promote locoregional control, the goal of adjuvant systemic therapy is to improve disease-free and overall survival by eradicating micrometastatic disease.^3^ In cases of large or locally advanced cancers, neoadjuvant systemic therapy may facilitate tumor shrinkage and “downstaging,” improved surgical outcomes, the ability to safely perform breast conservation, and an understanding of the sensitivity of the tumor to treatment.^4^

Although effective for treating breast cancer, systemic therapy also impairs key processes of normal cells. In particular, wound healing, immune response, and the coagulation cascade can be negatively affected. Therefore, it is of particular importance to consider the optimal choice and timing of systemic therapy in patients also undergoing surgery. Compounding this is the increasing rate of breast reconstructions, particularly immediately following mastectomy.^5^ Reconstructive surgeons have become integral to the breast cancer multidisciplinary team and must recognize the effects and implications of systemic therapy on breast reconstruction.^6^ The purpose of this article is to review the indications and common regimens of systemic therapy in early-stage breast cancer. Furthermore, the mechanism of action, approved uses, major toxicities, and relevance to breast reconstruction of specific agents are considered.

## METHODS

A review of the literature on PubMed and Cochrane databases was undertaken to define the indications and common regimens of systemic therapy in early-stage breast cancer. In addition, literature describing relevant information regarding specific systemic agents was reviewed. This article was also informed by the authors’ regular and ongoing interaction with numerous members of the multidisciplinary breast cancer treatment team at our institution. Such interactions occur frequently during the care of a patient with breast cancer, whether in clinic, at the bedside, and as part of multidisciplinary tumor board conferences. In these discussions, suggestions regarding appropriate treatments are made by each specialty, including medical oncologists, surgical oncologists, radiologists, radiation oncologists, pathologists, and plastic surgeons. After consideration of these suggestions in relation to a specific patient and her needs and desires, a consensus is reached on the most appropriate course. On the basis of this review and our own experience, we present here an overview of systemic therapy-based treatment strategies and recommendations for patients with early-stage breast cancer. Finally, we consider how such treatments may affect and be affected by breast reconstruction.

## RESULTS

### Chemotherapy

Chemotherapy has been a first-line breast cancer treatment for decades, as many large randomized trials have demonstrated its ability to prevent recurrence and prolong survival.^7^^,^^8^ Chemotherapeutic agents have a direct cytotoxic effect on cancer cells. The indications and 4 distinct classes of chemotherapy that are relevant to breast cancer treatment are presented.

#### Indications for adjuvant chemotherapy

The decision to offer adjuvant chemotherapy for early-stage breast cancer is based upon the absolute benefit and risk profile and is tailored to the individual patient. Several factors that are considered include stage, nodal status, receptor status, clinicopathology, and, more recently, genomic assay profiles. At the 2013 St Gallen International Breast Cancer Conference, an expert panel suggested that indications for adjuvant chemotherapy in early-stage breast cancer included triple-negative breast cancer, human epidermal growth factor receptor 2 (HER2)-positive disease, low hormone receptor status, high Ki-67 status, histologic grade 3 tumors, high 21-gene recurrence score, high-risk 70-gene signature, and the involvement of 3 or more lymph nodes (Table 2).^9^ In addition, some authors offer adjuvant chemotherapy for ER-positive, HER2-negative breast cancer for tumor size greater than 1 cm or if there is nodal involvement.^10^ The choice of chemotherapeutic regimen offered is tailored to the individual case. While national practice guidelines exist, the choice of a specific regimen is made by the treating oncologist (Table 3).^10^^,^^11^

#### Alkylating agents

Alkylating agents (Table 4) are one of the earliest classes of antineoplastic drugs and were identified by their similarity to nitrogen mustards in causing marked depletion of bone marrow and lymphoid tissue in exposed individuals.^12^ They impede cellular growth and induce apoptosis by cross-linking DNA.

The only member of the alkylating agents routinely used for breast cancer is cyclophosphamide. It is given in combination with other chemotherapeutic drugs and is typically used to decrease the risk of recurrence after surgery or to shrink large, advanced tumors prior to surgery. Cyclophosphamide not only is cytotoxic to highly proliferative cancer cells but can also injure normal cells with high turnover (eg, bone marrow, germ cells, gastric epithelium). Its biologic actions are dose-dependent and non–phase-specific.^13^

Adverse effects associated with cyclophosphamide use include malaise, nausea and vomiting, alopecia, amenorrhea, and stomatitis. Myocarditis and congestive heart failure have been observed in patients receiving high-dose cyclophosphamide (>2.4 g/m^2^).^14^ While this dose-dependent toxicity is generally not seen in patients receiving doses used for breast cancer, a feared dose-dependent complication that does have important implications for the surgical patient is myelosuppression. In patients receiving cyclophosphamide, the absolute neutrophil count typically reaches its nadir 10 to 12 days postadministration and recovers by 3 weeks.^15^ Thrombocytopenia and associated bleeding may also occur. Therefore, prior to any surgical intervention, preoperative laboratory testing should confirm that blood indices have returned to normal.

With respect to wound healing, studies of high-dose cyclophosphamide administration demonstrate impaired tensile strength^16^ and attenuated neovascularization during the proliferative phase of healing.^17^ These are most pronounced when cyclophosphamide is administered 24 to 48 hours postoperatively.^18^ However, in human trials, administration at doses that are consistent with standard breast cancer treatment doses does not increase wound healing complications.^19^ Therefore, if standard dosing is to be employed, the surgeon should not expect cyclophosphamide to impair healing or promote wound complications. In the case that extremely high doses are offered, no formal recommendations exist guiding timing of surgery related to chemotherapy.

#### Antimetabolites

Antimetabolites impair cancer growth by substituting for the normal precursors of RNA and DNA and disrupt nucleic acid synthesis (Table 4). These agents exert their effects during the S phase of the cell cycle, when the chromosomes are being copied. Antimetabolite medications that are routinely used for breast cancer treatment are methotrexate and 5-fluorouracil.

Major toxicities associated with antimetabolites include myelosuppression, mucositis, and nausea and vomiting. Methotrexate is also associated with hepatotoxicity and pulmonary toxicity. Prior to surgery, patients who have received antimetabolites should demonstrate normal blood indices via blood testing. In addition, if patients demonstrate pulmonary symptoms, a preoperative radiograph may be beneficial. Folic acid supplementation is also indicated for patients receiving methotrexate to reduce the risk of several of its adverse effects.

Of particular importance to patients undergoing surgery are reports that both methotrexate^20^ and 5-fluorouracil^21^ are associated with decreased wound tensile strength. The degree of wound impairment, however, remains unclear. One study focusing on 5-flurouracil demonstrated an increase in wound morbidity when treatment occurred 7 to 10 days postoperatively.^21^ To minimize these potential complications, it is therefore advisable to wait at least 14 days after surgery before administration of antimetabolite agents.

#### Anthracyclines

Anthracyclines are cytotoxic antibiotics derived from *Streptomyces* species that mediate anticancer effects by several mechanisms (Table 4).^22^ They prevent cell replication by intercalating between DNA and RNA base pairs (ie, inhibiting nucleic acid synthesis), by the inhibition of topoisomerase II that blocks DNA transcription, and by the generation of free oxygen radicals that damage DNA, proteins, and the cell membrane. Anthracyclines used for early-stage breast cancer include doxorubicin and, less commonly, epirubicin.

Adverse effects of anthracycline administration include nausea, vomiting, and alopecia. Major toxicities include cardiotoxicity and myelosuppression. Extravasation of doxorubicin can also cause localized tissue necrosis.

Anthracycline-induced cardiotoxicity can occur acutely with administration or remotely long after cessation of the drug. Although several risk factors exist, this side effect is largely related to cumulative dose. Prior to surgery, patients who have received treatment containing an anthracycline-based drug should undergo electrocardiography and echocardiography to assess cardiac fitness. In addition, anthracycline-related myelosuppression manifests as neutropenia, thrombocytopenia, and/or anemia. Blood work should be performed preoperatively to ensure return to normal values.

Doxorubicin administration has also been linked to impaired wound strength when administrated preoperatively,^23^ perioperatively,^18^ or postoperatively.^24^ It primarily affects the early phases of healing,^25^ and its effect on macrophage dysfunction may also delaying the healing process.^26^ On the basis of the available data, it is advisable to avoid doxorubicin administration 7 days prior to and after surgery.

#### Antimicrotubule agents

Taxanes are antimicrotubule agents that prevent microtubule disassembly within the nucleus of cancer cells, disrupting mitosis, cell division, and proliferation (Table 4).^27^ Taxanes that are often used for early-stage breast cancer treatment include paclitaxel and docetaxel. Because of the results of several powerful trials, these agents have become the most widely used in early-stage breast cancer in both the adjuvant and neoadjuvant settings.^28^^,^^29^

A significant side effect of taxanes is myelosuppression, particularly in patients with hepatic impairment.^30^ Therefore, prior to surgical intervention, normal blood counts must be ensured. Additional adverse effects include peripheral neuropathy, skin desquamation and erythema, alopecia, and nausea and vomiting. Hypersensitivity reactions may occur and can be prevented by premedication with histamine antagonists and oral corticosteroids.^31^ Data on wound healing and taxanes are sparse. One animal study assessing intrawound administration of docetaxel did not reveal any wound healing complications.^32^ No guidelines exist regarding surgery timing relative to taxane use.

### Hormonal therapy

Exposure to estrogen is a recognized risk factor for breast cancer development. In ER-positive disease, estrogen stimulates tumor genesis by upregulating mammary tissue proliferation,^33^ through direct genotoxic activity,^34^ and by the facilitation of oncogenic mutations.^35^ A primary goal of therapy in ER-positive disease is to reduce estrogen activity. Early approaches focused on surgical ablation of ovarian tissue. Despite positive results, these procedures were associated with significant and irreversible morbidity. Therefore, pharmacologic approaches have become a mainstay of therapy in receptor-positive breast cancer (Table 5).^36^

#### Tamoxifen

Tamoxifen is a selective estrogen receptor modulator (SERM) that inhibits breast cancer cell growth by competitively inhibiting estrogen from binding to its receptors in mammary tissue (Table 4). Transforming growth factor-beta (TGF-β) synthesis is upregulated in the presence of tamoxifen, which reduces mammary epithelial proliferation.^37^ In addition, tamoxifen causes decreased circulation of breast cancer mitogens.^38^ It is the hormonal agent of choice for the adjuvant treatment of premenopausal women with early-stage breast cancer, as well as postmenopausal women who are not candidates for aromatase inhibitors (AIs). In general, newly diagnosed pre- or perimenopausal women take tamoxifen for 5 years. Women who remain premenopausal receive tamoxifen for an addition 5 years, whereas postmenopausal women may receive tamoxifen for 5 more years or an AI for 5 years. In contrast, postmenopausal women who are newly diagnosed with hormone-positive breast cancer will choose from several options to best fit their individualized needs: tamoxifen for 10 years; an AI for 5 years; tamoxifen for 5 years, followed by an AI for 5 years; or tamoxifen for 2 to 3 years, followed by an AI for 5 years.

Common adverse effects of tamoxifen include transient menopause-like symptoms, a small increase in the risk of low-grade endometrial carcinoma,^39^ and an increased risk of venous thromboembolism (VTE).^40^ Advanced age and concomitant chemotherapy administration can further increase the risk of VTE to 2.5 times compared with placebo.^41^ The majority of thromboembolic events occur within 3 months of surgery or during a time of prolonged immobility.

Animal studies have revealed that tamoxifen is associated with increased intimal wall thickness of microvasculature.^42^ Furthermore, human studies have reported a significant increase in microvascular complications during autologous reconstruction in the setting of tamoxifen.^43^^,^^44^ It is therefore suggested that tamoxifen be discontinued for several days to weeks prior to surgery and restarted only after the elevated risk of VTE has resolved.^45^ Of note, tamoxifen may delay wound healing as noted in one retrospective study that demonstrated prolonged axillary drainage after primary breast cancer resection in patients receiving tamoxifen perioperatively compared with not.^46^ However, data is sparse and weak, requiring further prospective studies to better evaluate wound healing and tamoxifen.^47^

#### Aromatase inhibitors

Aromatase inhibitors reduce estrogen levels by the inhibition of aromatase, an enzyme that catalyzes the conversion of androgen to estrogen in adipose tissue (Table 6). In postmenopausal women, estrogen is predominantly produced from the conversion of androgens as the ovaries are no longer active. Therefore, while AIs are effective in postmenopausal women, they are of little value in premenopausal women. Three third-generation AIs (exemestane, letrozole, and anastrozole) are approved for breast cancer treatment in the United States.

Aromatase inhibitors are preferred to SERMs for the treatment of early-stage, ER-positive cancer in postmenopausal women because of superior efficacy in both the neoadjuvant and adjuvant settings.^48^^,^^49^ Also, they do not increase the risk of VTE or endometrial cancer, but they are associated with an increased risk of musculoskeletal pathology, cardiovascular disease, and hyperlipidemia.^50^ Although in vivo evidence suggests that AIs may impair wound healing, this has not been substantiated clinically.^51^ Presently, it is not recommended to discontinue AI therapy in the perioperative period.

### Targeted therapy

Biologic or targeted therapy entails the use of substances derived from living organisms or synthetic analogues to specifically target molecular markers. As more information regarding the genetic changes in cancer cells is uncovered, therapies directed at these changes are developed. Owing to their improved ability to target specific cells, targeted therapies are often associated with less severe adverse effects than other forms of systemic therapy (Table 7).^52^

#### Trastuzumab

Both HER2 gene amplification and HER2 receptor overexpression are found in approximately 25% of invasive breast cancers and are associated with a more aggressive clinical phenotype.^53^ The first agent approved for HER2-positive breast cancer was trastuzumab, which is a humanized monoclonal antibody. By binding to and preventing activation of an intracellular tyrosine kinase domain on the HER2 receptor, trastuzumab stimulates cell-cycle arrest and apoptosis in HER2-positive cells.^54^ Large, well-designed trials have demonstrated a survival benefit with trastuzumab administration in both early and advanced HER2-positive breast cancer, and it is now standard of care in HER2-positive disease.^55^^,^^56^

Although well tolerated, trastuzumab may cause influenza-like symptoms, nausea, and diarrhea. In addition, 4% of patients will experience cardiac toxicity ranging from an asymptomatic ejection fraction decrease to clinical heart failure.^57^ This risk increases when administered concurrently with various chemotherapeutic drugs. Prior to an operative procedure, patients receiving trastuzumab should undergo assessment of cardiac function. Trastuzumab has not been associated with poor wound healing.^58^

#### Emerging agents for targeted therapy

Pertuzumab is a monoclonal antibody that belongs to the class of HER dimerization inhibitors. It thwarts cancer cell growth by binding to HER2 and inhibiting dimerization of HER2 with other HER receptors, a process that is necessary for critical cell signaling.^59^ Pertuzumab is approved for use in combination with trastuzumab and docetaxel in patients with HER2-positive metastatic breast cancer. It has also been approved in the neoadjuvant setting of HER2-positive inflammatory, locally advanced, and early-stage breast cancer.^60^ Because of promising results, significant antitumor activity, and a safe toxicity profile associated with pertuzumab in neoadjuvant trials, it is currently under evaluation in the adjuvant setting of patients with operable HER2-positive breast cancer (NCT01358877).

Ado-trastuzumab emtansine (T-DM1) is a novel anti-HER2 therapy that consists of an antibody-drug conjugate in which trastuzumab is linked to the microtubule inhibitor DM1. It is approved for use in metastatic breast cancer and is under investigation in the adjuvant (NCT01772472 and NCT01966471) and neoadjuvant (NCT02131064) settings. Although not approved in the perioperative setting, the results of early phase trials are promising. Additional agents that have demonstrated success in the metastatic setting and are currently under investigation as adjuncts or alternatives to approved targeted agents in the adjuvant setting include lapatinib and palbociclib. Further investigation is warranted to determine which patients would benefit from specific targeted therapies and the associated adverse effects.

### Neoadjuvant therapy

The primary goal of neoadjuvant therapy is to improve surgical outcomes in patients for whom primary surgery is technically challenging or not feasible (eg, advanced disease) or for patients who desire breast conservation but require a mastectomy or a partial mastectomy that would result in a poor cosmetic outcome.^61^ In addition, neoadjuvant therapy facilitates early evaluation of treatment effectiveness, allowing for earlier discontinuation of ineffective treatment. Recent evidence also suggests that patients who are poor responders to neoadjuvant therapy derive benefit from further systemic therapy in the adjuvant setting.^62^ Patient selection is based upon operability, clinical status of the patient, and the characteristics of the cancer (Table 8).^63^^,^^64^ Patients likely to have a positive locoregional response (eg, HER2-positive and triple-negative breast cancer) are better suited to neoadjuvant therapy.^65^ Although associated with high rates of clinical response and improved surgical outcomes, neoadjuvant therapy does not provide a mortality benefit compared with adjuvant therapy.^66^^,^^67^

### Systemic therapy and breast reconstruction

Importantly, breast reconstruction, whether performed in immediate or delayed fashion, does not negatively impact oncologic outcomes. There have been sporadic reports of a small but significant delay in time to chemotherapy after immediate breast reconstruction^68^; however, these results were not clinically significant and other authors have reported no delay.^69^ In addition, when the recommendations herein are followed, adjuvant chemotherapy after breast reconstruction does not increase the risk of reconstructive failure,^70^ surgical or wound healing complications,^71^ or poor cosmesis.^72^

With respect to neoadjuvant chemotherapy, early reports suggested that it may be associated with increased donor site complications and fat necrosis after autologous breast reconstruction^73^ and a high proportion of failure in expander-based reconstruction.^74^ However, more recent evidence suggests that complication rates following immediate reconstruction are not elevated.^75^ A 2014 meta-analysis of 11 studies found that neoadjuvant chemotherapy did not increase complications after immediate breast reconstruction when infection, hematoma, seroma, expander or implant loss, and flap loss were considered.^76^ In addition, a prospective evaluation of skin flap necrosis after mastectomy and reconstruction showed that many factors, but not neoadjuvant chemotherapy, were not associated with flap necrosis.^77^ Therefore, there is compelling evidence that complications rates associated with appropriately performed breast reconstruction are not increased because of chemotherapy. To optimize outcomes, it is prudent to allow at least 6 to 8 weeks after the completion of neoadjuvant chemotherapy for the recovery of bone marrow and other physiologic processes prior to surgery.^14^

Much less studied are the specific effects of hormonal or targeted therapy on breast reconstruction. Although tamoxifen may increase the risk of microvascular flap complications,^43^ its use has not been shown to increase the risk of other complications including the need for blood transfusion.^78^ However, because of its effect of thrombosis and wound healing, tamoxifen should not be used perioperatively. There are currently no reports in the literature describing complications associated with tamoxifen after breast reconstruction. Because of the potential for cardiac complications and the large number of patients taking trastuzumab who present for elective breast reconstruction, it would be beneficial to study the effect of trastuzumab on the perioperative period in this population.^79^

## DISCUSSION AND RECOMMENDATIONS

The treatment of breast cancer has evolved significantly over the past half century. In addition to primary surgical therapy and radiation therapy, systemic forms of therapy are frequently offered for early-stage disease. Therefore, an understanding of their effects on normal physiologic processes is crucial. In addition, abiding by data-driven recommendations regarding their use minimizes the potential complications associated with surgical procedures.

The following recommendations should be considered when offering reconstruction to patients who have or will receive chemotherapy, hormonal therapy, and/or targeted therapy as part of their care:

1. The plastic surgeon should be present for discussions of the overall care plan with the patient and other members of the multidisciplinary treatment team.
2. Ensure adequate time for the healing of surgical sites before the initiation of adjuvant chemotherapy.
3. Ensure adequate time between the completion of neoadjuvant chemotherapy and surgery.
4. Prior to any surgical procedure, ensure that required laboratory, radiologic, and clinical tests have been reviewed on the basis of the agents received by the patient.
5. Determine whether the patient can safely undergo expander/implant-based and/or autologous-based reconstruction in light of the agents received and follow-up test results (eg, Do patients who have received cardiotoxic agents demonstrate the cardiac fitness to undergo a long, complex procedure?).
6. Ensure discontinuation of tamoxifen 2 or more weeks to 4 weeks prior to surgery and resume only after the elevated thromboembolic risk has resolved.
7. Remain educated on recent literature and the latest recommendations regarding the use of systemic agents in patients undergoing breast reconstruction

## Figures and Tables

**Table 1:** Anatomic stage/prognostic groups for early-stage breast cancer*

Stage	Primary tumor (T)	Regional lymph nodes (N)	Distant metastasis (M)
IA	T1	N0	M0
IB	T0	N1mi	M0
	T1	N1mi	M0
IIA	T0	N1	M0
	T1	N1	M0
	T2	N0	M0
IIB	T2	N1	M0
	T3	N0	M0
IIIA	T0	N2	M0
	T1	N2	M0
	T2	N2	M0
	T3	N1	M0
	T3	N2	M0

*T0 indicates no evidence of primary tumor; T1, tumor ≤20 mm in greatest dimension; T2, tumor >20 mm but ≤50 mm in greatest dimension; T3, tumor > 50 mm in greatest dimension; N0, no regional lymph node metastasis; N1mi, micrometastases to movable ipsilateral level I, II axillary lymph node(s); N1, metastasis to movable ipsilateral level I, II axillary lymph node(s); N2, metastasis to ipsilateral level I, II axillary lymph nodes that are clinically fixed or matted; or in clinically detected ipsilateral internal mammary nodes in the absence of clinically evident axillary lymph node metastases; and M0, no clinical or radiographic evidence of distant metastases.

**Table 2:** Indications for adjuvant chemotherapy in early-stage breast cancer*

Triple-negative
HER2-positive
Low hormone receptor status
High Ki-67 status
Histologic grade 3 tumors
High 21-gene recurrence score
High-risk 70-gene signature
Involvement ≥3 lymph nodes
ER-positive, HER2-negative^†^

*From references Goldhirsch et al^9^ and Burkard et al.^10^

†If tumor size >1 cm or there is lymph node involvement.

**Table 3:** Common regimens of adjuvant chemotherapy in early-stage breast cancer*

Indication	Regimen	Cycle length, d	Total cycles	Monitoring
HER2-negative^†^	TC^‡^	21	4	CBC every cycle; CMP/LFT every 2 wk
	Dose-dense TC^‡^	14	4	
	ACT^§^	21 (AC)	4 (AC)	CBC/CMP/LFT every 2 wk; assess neurologic function prior to paclitaxel
		7 or 21 (T)	12 or 4 (T)	
	Dose-dense ACT^§^	14 (AC)	4 (AC)	
		7 or 14 (T)	12 or 4 (T)	
	AC^‖^	21	4	CBC/CMP/LFT every 2 wk
	TAC^¶^	21	6	CBC/CMP/LFT every 3 wk
	CMF**^#^**	28	6	CBC every cycle; CMP/LFT prior to therapy
HER2-positive**	ACTH^††^	21 (AC)	4 (AC)	CBC/CMP/LFT every cycle (ACT); assess neurologic function prior to paclitaxel; assess cardiac function prior to and during trastuzumab administration
		7 (TH)	12 (TH)	

*From references Burkard et al^10^ and Brenner et al.^11^ CBC indicates complete blood count with platelets; CMP, complete metabolic panel (serum electrolytes, renal function tests); and LFT, liver function tests.

†TAC is used for node-positive disease; all other regimens are used in node-negative disease.

‡Docetaxel and cyclophosphamide (TC); dose-dense TC is infrequently used.

§Doxorubicin and cyclophosphamide (AC), followed by paclitaxel (T); for ACT, paclitaxel can be administered weekly for 12 weeks or once every 3 weeks for 4 cycles. For dose-dense ACT, paclitaxel can be administered every 14 days for 4 cycles or in standard fashion (weekly for 12 weeks).

‖Doxorubicin and cyclophosphamide (AC)

¶Docetaxel, doxorubicin, and cyclophosphamide (TAC)

#Cyclophosphamide, methotrexate, 5-fluorouracil (CMF).

**Trastuzumab is added in HER2-positive disease. Other commonly used trastuzumab-based regimens for HER2-positive disease include paclitaxel and trastuzumab; docetaxel, carboplatin, and trastuzumab; and paclitaxel, carboplatin, trastuzumab, and pertuzumab.

††Doxorubicin and cyclophosphamide (AC), followed by paclitaxel (T) and trastuzumab (H).

**Table 4:** Chemotherapeutic agents used in the treatment of early-stage breast cancer*

Class	Drug	Mechanism of action	Adverse effects relevant to breast reconstruction^†^	Evidence-based recommendations
Alkylating agents	Cyclophosphamide	Cross-links DNA	Myelosuppression	Check CBC and cardiac fitness prior to surgery^‡^
			Cardiotoxicity	
Antimetabolites	5-Fluorouracil	Prevents thymidine synthesis	Myelosuppression	Check CBC prior to surgery^‡^; avoid administration within 14 d of surgery
			Impaired wound healing	
	Methotrexate	Prevents tetrahydrofolic acid synthesis	Myelosuppression	Check CBC/LFT/CXR prior to surgery^‡^; avoid administration within 14 d of surgery
			Impaired wound healing	
			Hepatotoxicty	
			Pulmonary toxicity	
Anthracyclines	Doxorubicin	Intercalates DNA	Myelosuppression	Check CBC and cardiac fitness prior to surgery^‡^; avoid administration within 7 d of surgery
		Inhibits topoisomerase II	Cardiotoxicity	
			Impaired wound healing	
	Epirubicin	Intercalates DNA	Myelosuppression	Check CBC and cardiac fitness prior to surgery^‡^
		Inhibits topoisomerase II	Cardiotoxicity	
Antimicrotubule agents	Paclitaxel	Prevents microtubule disassembly	Myelosuppression	Check CBC prior to surgery^‡^
	Docetaxel	Prevents microtubule disassembly	Myelosuppression	Check CBC prior to surgery^‡^

*CBC indicates complete blood count with platelets; LFT, liver function tests; and CXR, chest radiographs.

†Common and/or significant toxicities with respect to patients undergoing surgical procedures are noted.

‡When drug is used in neoadjuvant setting.

**Table 5:** Indications for adjuvant hormonal therapy in early-stage breast cancer*

All patients with hormone receptor-positive breast cancer
Premenopausal
Tamoxifen (all women)
Ovarian suppression plus tamoxifen or aromatase inhibitor (<40 y of age or high-risk)
Aromatase inhibitors should not be used in women with intact ovarian function
Postmenopausal
Aromatase inhibitor preferred (tamoxifen acceptable if aromatase inhibitor not tolerated or contraindicated)^†^
Extended aromatase inhibitor if node-positive (still controversial)

*From references Goldhirsch et al^9^ and Pritchard.^36^

†For women who start on tamoxifen, it is recommended to switch to an aromatase inhibitor after 2 years if they become postmenopausal during that time. If a 5-year course of tamoxifen has been completed, an additional 5-year course of an aromatase inhibitor may be recommended.

**Table 6:** Hormonal agents used in the treatment of early-stage breast cancer*

Class	Drug	Mechanism of action	Adverse effects relevant to breast reconstruction^†^	Evidence-based recommendations
SERM	Tamoxifen	Inhibits estrogen binding at mammary tissue	VTE	Avoid administration several days to weeks perioperatively^‡^
			Impaired wound healing	
AI	Exemestane	Irreversibly inhibits aromatase	Cardiotoxicity	Consider cardiac fitness evaluation prior to surgery^§^
	Letrozole	Reversibly inhibits aromatase	Cardiotoxicity	Consider cardiac fitness evaluation prior to surgery^c^
	Anastrozole	Reversibly inhibits aromatase	Cardiotoxicity	Consider cardiac fitness evaluation prior to surgery^c^

*AI indicates aromatase inhibitor; SERM, selective estrogen receptor modulator; and VTE, venous thromboembolism.

†Common and/or significant toxicities with respect to patients undergoing surgical procedures are noted.

‡Restart after surgery only after elevated risk of VTE has normalized (may be ≥4 weeks).

§When drug is used in neoadjuvant setting.

**Table 7:** Indications for adjuvant targeted therapy in early-stage breast cancer*

HER2-positive, node positive
HER-positive, node-negative, tumor ≥0.5 cm
HER-positive, node-negative, tumor ≥0.3 cm, high-risk features^†^

*From references Goldhirsch et al^9^ and Burstein.^52^

†High-risk features include hormone-receptor negative. This criterion is controversial.

**Table 8:** Indications for neoadjuvant therapy*

Systemic therapy	Indications^†^
Chemotherapy	• TNBC (nonoperable or large tumor and BCS desired) • Hormone-receptor positive, HER2-negative (nonoperable or large tumor and BCS desired) • HER2-positive (nonoperable or large tumor and BCS desired)
Hormonal	• Postmenopausal, strong hormone receptor positivity, low proliferating disease • Women who refuse or are not suited for NACT and initial surgery^‡^
Targeted	• Patients with HER2-positive disease who are receiving NACT

*From Goldhirsch et al,^9^ Sikov and Wolff,^63^ and Gradishar.^64^ BCS indicates breast-conserving therapy; NACT, neoadjuvant chemotherapy; and TNBC, triple-negative breast cancer.

†Inoperable breast cancers include inflammatory breast cancer, bulky or matted N2 axillary nodes (ie, metastasis in ipsilateral level I or II axillary lymph nodes), N3 nodal disease (ie, metastasis in ipsilateral level III axillary lymph nodes), and T4 tumors (ie, direct extension to chest wall and/or skin).

‡In this population, there is little data regarding the benefits, risks, and outcomes of this approach.
